# Staff perceptions of factors affecting the use of RAS-DS to support collaborative mental health practice

**DOI:** 10.1186/s12888-023-04996-2

**Published:** 2023-07-12

**Authors:** Anne Honey, Nicola Hancock, Justin Newton Scanlan

**Affiliations:** School of Health Sciences, The University of Sydney, Susan Wakil Health Building (D18), Camperdown, NSW 2006 Australia

**Keywords:** RAS-DS (Recovery Assessment Scale: domains and stages), Recovery-oriented practice, Consumer empowerment, Measurement, Person-centred services

## Abstract

**Background:**

The Recovery Assessment Scale: Domains and Stages (RAS-DS) was designed to be both a recovery outcome measure and a tool to enhance service-user control over their recovery journey. While extensively and globally used in mental health services for the former purpose, routine use for the latter purpose is yet to be realised. The aim of this study was to identify barriers, facilitators and additional supports needed for RAS-DS to be used to support service user participation, goal setting and recovery action planning.

**Methods:**

An online survey was conducted of mental health workers who had engaged with RAS-DS, including fixed choice and open-ended questions. Data were analysed using descriptive statistics and interpretive content analysis respectively.

**Results:**

The 65 respondents reported more frequent use of RAS-DS as an outcome measure than as a collaboration tool and more than half reported difficulties in using it in this way. Factors that they described as influencing the use of RAS-DS as a tool for collaboration and support included: previous experiences with RAS-DS; organisational supports and policies; awareness of the RAS-DS amongst colleagues; RAS-DS related training and support; staff time and capacity; the format of RAS-DS; service user population or context; and respondents’ own active efforts.

**Conclusions:**

Extending the use of RAS-DS, an already widely used tool, to routinely support recovery-oriented practice has both efficiency and service user empowerment benefits. However further work is needed to enable this including: provision of co-designed, accessible training resources; a user platform including built in guidance; and strategies to promote management understanding and valuing of the enhanced recovery-orientation opportunities inherent in RAS-DS use.

## Introduction

Worldwide, mental disorders are among the top ten leading causes of burden of disease [1]. Over 3 million Australians each year seek professional help for their mental health [2]. Severe, persistent, and complex mental illness is experienced by approx. 625,000 Australians with around 65,000 having a resulting psychosocial disability [3]. These people, like other members of society, have a right to choice and self-determination [4]. Mental health consumer activists, reform advocates and policy makers have long called for greater service user self-determination and control over the mental health services and supports they use [5]. Increasingly there is policy-level consensus across English-speaking countries around the importance of adopting a recovery-oriented approach to mental health service delivery [6]. Recovery in this context does not refer to cure, but to people living with mental illness “regaining control of their identity and life, having hope for their life, and living a life that has meaning for them” [4, p.5]. A recovery-oriented approach demands that the needs and priorities of individual service users drive service provision, rather than organisational or staff priorities dictating the intervention focus. In this approach, service users are supported to take control and leadership of identifying their needs and goals, planning the steps to recovery, and selecting and directing the services and supports they use.

Evidence increasingly demonstrates that having a greater influence over recovery related decision-making can improve health-related outcomes for mental health service users. This is supported by findings from several recent studies [e.g., 7–9] including a longitudinal study involving 588 patients with severe mental illness [10]. These studies found that greater service user involvement in, and control over, clinical and recovery-focused decisions was associated with: increased treatment engagement and motivation; improved social functioning; quality of life; reduced symptom severity; and lower illness burden associated with symptoms, interpersonal difficulties, and problems in social roles.

Despite the evidence and policy mandates for service user control, practice implementation has lagged. While shared decision-making is recommended as an approach to enhance self-determination and choice [11], a recent review of shared decision-making in mental health contexts evidenced the dearth of application beyond the realm of medication-focussed decisions [12]. Multiple reasons have been given for this lack of implementation including clinicians’ paternalistic views and stigma related beliefs, fear of litigation, cognitive impacts of mental illness, and internalised self-stigma [13, 14]. Thus many service users are not given the opportunity to drive their recovery journey. Goals and recovery action plans continue to be directed by staff or organisational values and perspectives rather than service user priorities [8, 15]. Although some health services use tools designed to develop wellness plans with service users [16], these tools are developed within individual organisations, often for service accountability purposes [17]. There is little evidence of the usefulness of these tools in personal recovery. They are not freely available, are divorced from self-assessment tools and are often not seen as meaningful by service users or staff [18]. In addition, they are generated by and hosted within individual health services, where they are not accessible to service users, which can compromise therapeutic alliance [18].

The Recovery Assessment Scale – Domains and Stages (RAS-DS) [19–21] was developed with mental health service-users [22–24] and is well validated through extensive testing [19–23, 25]. It was designed to fulfil two functions [26]. The first function is to measure self-reported outcomes. The second is to enhance service-user control over establishing intervention goals and recovery action plans.

As a self-report outcome measure, the RAS-DS is extensively employed. It is used in more than 26 countries across Europe, the United Kingdom, America, Canada, Africa, and Australasia and has been translated into 18 languages [www.ras-ds.net.au]. In Australia, where it was developed, it is the Federal Department of Health’s recommended self-report outcome measure for all psychosocial support programs. Further, it is widely adopted across state, non-government and private mental health services and organisations. It has been used and tested in Australian programs for adults and people in early psychosis intervention programs [19, 20].

Despite its global use as an outcome measure, routine concurrent use of the RAS-DS to enhance service user control is yet to be realised. While many clinicians and mental health workers report using it solely as an outcome measure [27], service users have indicated that they value its other function. As one service user noted: “it would be good to use this in goal setting” [19, 20]. This is a significant missed opportunity. Using RAS-DS to support service user driven goals, recovery action plans and interventions can support recovery-oriented practice and service user driven services. It also avoids creating additional workload for clinicians and assessment/administrative load for service users because it extends the use of a widely used outcome tool rather than introducing additional tools.

In summary, the potential benefits of using the RAS-DS as a collaborative planning tool to enhance service-user control over establishing intervention goals and recovery action plans are considerable. Yet its use in this way is relatively low. It is therefore critical to understand the reasons and circumstances that prevent mental health workers and organisations from using the RAS-DS as more than an outcome measure. Additionally, it is important to explore what supports would help them to do so. Therefore, this study explored the perspectives of mental health workers who use the RAS-DS. The aim was to understand the barriers, facilitators and additional supports needed for themselves and other mental health workers to use the RAS-DS as a tool to support greater service user participation in goal setting and recovery action planning.

## Method

To address the research aim, a descriptive survey design was adopted, using a mixture of qualitative and quantitative analysis. The University of Sydney Human Research Ethics Committee approved the study (Protocol # 2022/005).

### Sampling and recruitment

Participants were mental health workers who had engaged with the RAS-DS in practice. They were recruited from a database of people and organisations who had contacted the authors requesting to use RAS-DS and had also agreed to be contacted by the authors about RAS-DS related matters. Emails were sent to 306 individual workers and organisations. Recipients were provided with a Participant Information Statement and invited to participate in the anonymous online questionnaire. Two follow up emails were sent to maximise response rates.

### Data collection

An online questionnaire hosted on REDCap© [28] was used to collect data. The questionnaire contained three parts. The first asked participants about themselves, their role, and their work context. The second contained fixed response questions about how frequently people use RAS-DS, for what purposes, and areas of difficulty in using it as a tool for collaboration and support. The third consisted of four open-ended questions. These asked participants to reflect, in their own words, on barriers and enablers to using RAS-DS to support people to develop their own recovery goals and plans, supports that would help them, and any other comments.

### Data analysis

Descriptive statistics were used to analyse the quantitative data. Free text answers to the open-ended questions were analysed using interpretive content analysis (ICA). This is a hybrid research method that employs both qualitative and quantitative techniques [29, 30]. First, free text responses were imported into qualitative analysis software NVivo [31]. They were inductively coded using constant comparative analysis [32]. This systematic method uses line-by-line coding to identify the main idea within each segment of data, comparing each data segment with previously developed codes and grouping similar ideas together. This inductive approach allows the researcher to generate codes directly from the data rather than manipulating the data to fit into pre-determined categories. Responses were then re-examined to ensure that they were fully coded to the identified codes, and the number of individuals who discussed each concept was calculated [29].

## Findings

### Participants

We received 65 completed responses. Of these, 59 people responded to at least one of the open-ended questions. Table 1 provides a summary of characteristics and contexts of participants.

**Table 1:** Participant contexts

	N=65	%^a^
Area of work:		
Acute inpatient service/hospital Inpatient rehabilitation Community based service Other (e.g., private practice, outpatients, academic, sub-acute)	8 13 46 7	12.3% 20.0% 70.8% 10.8%
Age groups of service users:		
Children 0–12 Adolescents 12–18 Youth/early intervention 12–25) Adults 18–65 Older adults 65+	3 12 16 57 19	4.6% 18.5% 24.6% 87.7% 29.2%
Profession:		
Peer worker Allied health clinician Nurse Doctor/psychiatrist Other mental health worker Other (e.g., team leader/manager, administrator, activities coordinator, psychologist, social worker)	11 17 3 4 24 14	16.9% 26.2% 4.6% 6.2% 36.9% 21.5%
Country of practice:		
Australia United Stated Canada Singapore Other (1 each of France, Indonesia, New Zealand, Scotland, South Africa, South Korea, Sweden, Thailand)	33 12 10 2 8	50.8% 18.5% 15.4% 3.1% 12.3%

^a^ Respondents could select all options that applied, so totals may be greater than 100%

### Current uses of RAS-DS (n = 65)

**Fig. 1:**
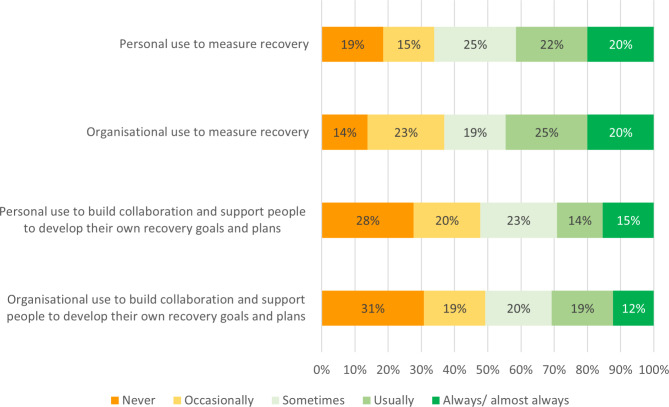
Frequency of personal and organisational use for measurement and collaboration

As expected, respondents in this study most frequently used the RAS-DS as an outcome measure and reported that their organisation used it as an outcome measure. Figure 1 shows frequency data for type of use. Only 15% of individuals and 12% of organisations always used the RAS-DS as a tool for collaborative goal setting and recovery planning. Respondents indicated that, while only 14% of organisations never used RAS-DS as an outcome measure, more than twice that number never used it as a collaborative tool.

### Difficulties experienced in using RAS-DS for collaboration and support to develop recovery goals and plans (n = 65)

**Fig. 2:**
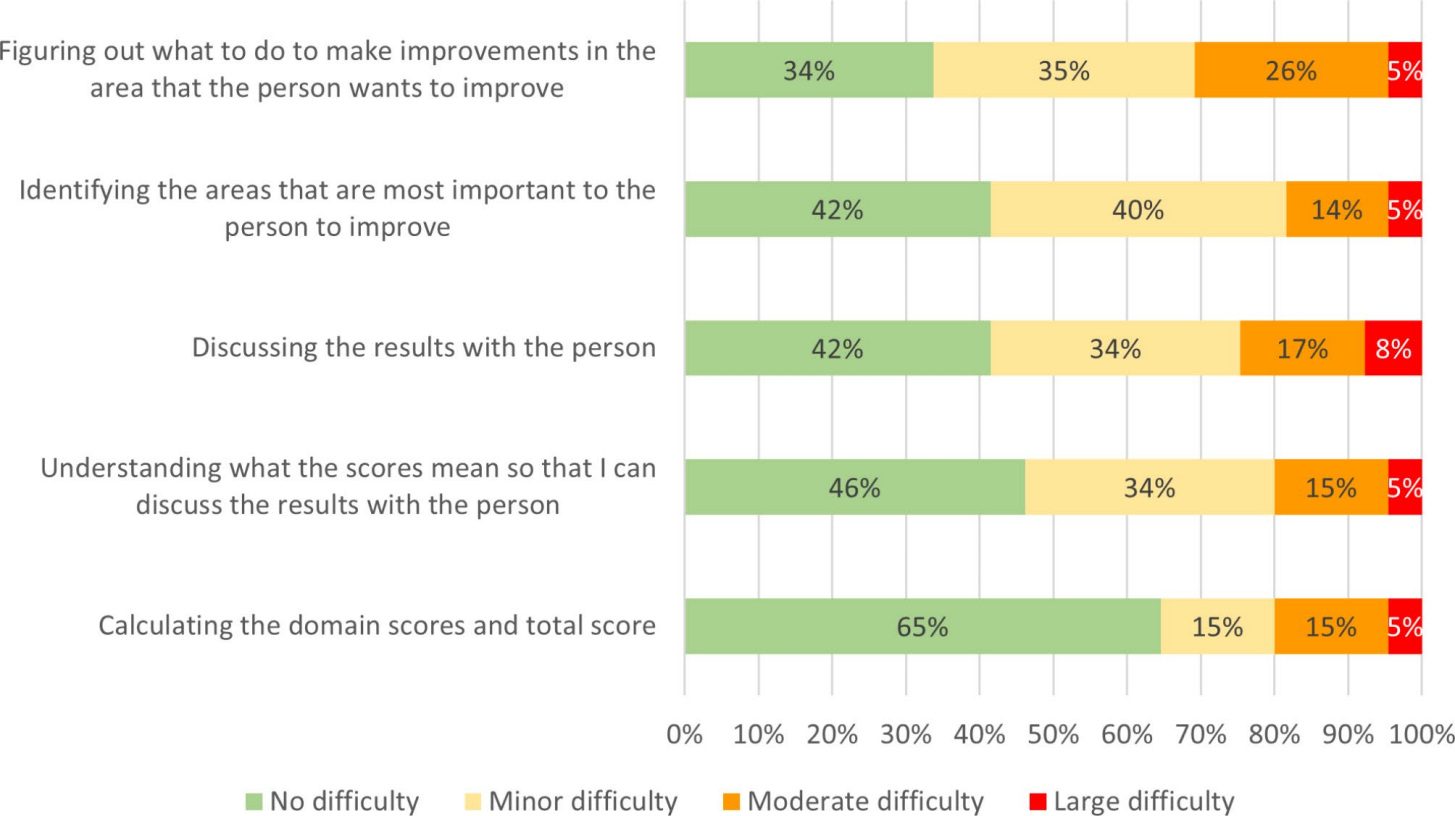
Difficulty experienced by respondents

Most respondents reported some difficulty in the areas specified. Figure 2 depicts the amount of difficulty reported for each aspect. With the exception of scoring the RAS-DS, more than half of respondents had some level of difficulty with all areas specified. Figuring out intervention strategies was the area respondents found most difficult, with nearly a third of participants experiencing moderate or large difficulty.

### Factors influencing use of RAS-DS as a tool for collaboration and support

In their free text answers, respondents added further depth. They discussed a number of issues that influenced their ability, or the ability of their organisation to use RAS-DS as more than an outcome measure, but as a tool to support collaboration and service user driven goal setting and recovery action planning. These included: their previous experiences with the RAS-DS; organisational supports and policies; awareness of the RAS-DS amongst colleagues; RAS-DS related training and support; staff time and capacity; the format of the RAS-DS; the service user population or context; and their own active efforts.

#### Previous experiences with RAS-DS (n = 40)

When asked about things that facilitated their use of RAS-DS as a goal setting tool and in other comments, 29 respondents replied simply that they had found it to be a positive, straightforward, and useful tool for that purpose, or that service users liked it.

*A good way to learn about individual consumers.*

*It helps participants to think about and reflect upon their recovery journey.*

*Using the four domains as a platform to engage in deeper meaningful conversation during the intake and service planning process helps to set the awareness of growth within the recovery journey. The RAS-DS is perfectly aligned to help an individual see (with support) the truth of their circumstances and build their growth one domain at a time.*

However, 24 respondents, including 14 who had also reflected on positive experiences with the tool (as above), mentioned features of their experiences of using RAS-DS that were less positive. Some reported having observed that certain service users: gave unreliable responses (e.g., just ticking anything) (n = 5); found it stressful or upsetting (n = 4); or struggled to understand it (n = 4). Others thought that it was too long (n = 3), or labour intensive to use and score (n = 3). A couple suggested that the language was not optimal (e.g., ‘mastering my illness’; n = 3) or would have liked to have different versions specific to their populations (e.g., older people, people with cognitive issues; n = 7).

#### Organisational supports and policies (n = 26)

Respondents talked about the importance of institutional supports and policies for use of RAS-DS. Institutional support included things like: making it part of mandatory data collection and standard processes and pathways; not having too many other requirements for assessments so that RAS-DS became an extra burden; organizing training; and allocating time for use of RAS-DS.

*[What is needed is] for this tool to be embraced at a systems level by upper management.*

Six people reported that institutional supports or policy facilitated their use of RAS-DS, while 20 people considered it a barrier.

*We require all Peer Supports to use the RAS-DS as their monthly tracking with the individuals they work with in two ways. One as a tracker of the individuals’ progression through recovery and two as the foundation to creating a recovery plan.*

*The [organisation] I work for uses way too may assessment scales including the RAS DS. It’s just another one I use because I have to.*

Three respondents noted that they worked in organisations that had not used RAS-DS in the past, but they were hoping to change that, while four more reported that a significant barrier to using it was an overall lack of recovery orientation within their service.

*[What is needed is] if you could convince the [organisation] that measuring recovery is important*

#### Awareness of RAS-DS amongst colleagues (n = 12)

Closely related to institutional and policy support was the knowledge of RAS-DS amongst respondents’ colleagues and their understanding of its potential. While one respondent mentioned that having other colleagues who were interested in using RAS-DS was a facilitator to its use, ten said that colleagues’ lack of awareness of RAS-DS was a barrier. They discussed the need for resources to promote knowledge about RAS-DS as a recovery-supporting tool among staff, including promotional resources and case studies emphasising the benefits.

*Clinical staff in the agency in which I work have not been introduced to the RAS-DS*

*A YouTube clip [would be helpful] that staff can easily access to understand why to use the form, what the benefits are and how it can be best used would be a great tool to easily share with colleagues, as basic training. Because the assessment is pretty self-explanatory, it would be more about raising awareness of the form and building an understanding of what the benefits of using it are both as a worker and as a client.*

Two respondents talked about the specific need for evidence that the tool was useful for collaborative goal setting, or more useful than alternative tools.

*In order to suggest that this tool be used for this purpose, it would be helpful to have the knowledge in how using the RAS-DS for helping clients develop recovery goals and plans compares to other tools currently in use for this process.*

#### RAS-DS related training and support (n = 34)

Beyond being aware of and understanding the benefits of using RAS-DS as a recovery-oriented goal setting tool, many respondents discussed the importance of knowledge and training, for both themselves and others, about how to use the RAS-DS in this way. Nine people thought lack of this knowledge and training was a barrier.

*Some clinicians cannot figure out how to structure a session around the RAS-DS.*

*I think early on however, many of us found it difficult to know what to do with the numbers/scores that we get, once we’ve completed it. And trying to explain what it really means.*

Four people said their use of RAS-DS had been facilitated by receiving training (n = 3) or watching “the videos re how to use it”. Most, however (n = 29), discussed the potential for additional support to help them and other people to use RAS-DS as a collaborative recovery-focused tool. This included: general training (n = 12); resources such as videos and written materials containing guidelines and practical examples (n = 18); and a community of practice (n = 2).

*I need some training with my colleagues on how to use the RAS-DS beyond them just filling it out.*

*Providing some hypothetical examples of completed RAS-DS’s and what goals they might imply for the person may be helpful*

#### Staff time and workload (n = 13)

As well as understanding the benefits and knowing how to use it as a collaborative recovery-planning tool, respondents reported that staff also needed enough time and space to spend with service users to use the RAS-DS this way. Due to high workloads, complex caseloads, and other assessment requirements, 12 of the 13 respondents that discussed this theme said that staff time, availability and “brain-space” were barriers to them using the RAS-DS beyond an outcome measure.

*The crunch on staff time ... We do not have the staff time to commit to reviewing data with individual clients to look at recovery goals*

*What prevents us is getting caught up in the complexities of each of our cases and having so much to think about when we receive a referral.*

This is related to institutional support and policy, where institutional demands, different priorities and high workloads inhibit time spend on person focused goal setting.

#### The format of the RAS-DS (n = 7)

Seven respondents commented on how the RAS-DS format contributed to the ease of using it for goal planning. Two appreciated their organisations providing a digital format and three more suggested that having RAS-DS in the form of a digital app would be useful. Two recommended providing a resource to simplify the scoring process and that would enable it to be completed “out in the field” so that results could be immediately discussed with service users. Two suggested the inclusion of specific tools to foster goal setting.

*Having access to an easy/straightforward tool that supports the exercise of incorporating the RAS-DS into goal planning.*

*Some paper/online tools that perhaps pre-fill in the results of the RAS-DS into a goal setting format or care plan format.*

#### The service-user population or context (n = 11)

Five respondents felt that using RAS-DS as a tool for collaboration and recovery planning was unnecessary within their contexts. While one asserted that their service users did not usually need help to identify their goals, four reported that they used other methods to develop recovery goals.

*We have other tools and measures that can assist people to track their progress over time and allow them to develop specific goals for their personal recovery (e.g. Strengths Assessment Tool, Individual Recovery Plan).*

Seven respondents felt that using RAS-DS for goal setting was inappropriate, impractical, or prohibitively difficult in their specific settings or situations. These included: short term services where service users get lost to follow-up (n = 5); service users with predominantly negative symptoms (n = 1); and homeless service users (n = 1).

#### Their own active efforts (n = 15)

A number of respondents talked about things that they had done or could do to facilitate or support the use of RAS-DS as a collaborative recovery-planning tool. These included: making it part of their routine (n = 5); integrating it or linking the findings with other tools used (n = 4); ensuring the comfort of the service user through “chatting with clients, building rapport and providing comfortable/safe space” as well as being flexible, such as providing options for completion (n = 3); promoting RAS-DS within the workplace (n = 3); and prioritising the time and space to actually do RAS-DS with service users.

*Just keeping it in mind in our weekly planning meeting on a Monday. Bringing it up in operational meetings with the team extolling the benefits of it.*

*Probably a specific earmarking of time for staff to set aside and focus on the tool and using the information from it to review with patients. I don’t think special instructions are needed, more a commitment on our part to start using it more deliberately.*

## Discussion

Enhanced service user self-determination and self-efficacy is a human rights-based and evidence-based international priority in mental health [33–35]. However, shifting systems towards adopting more person-centred and shared decision-making approaches has been slow. Using recovery focused outcome measures such as RAS-DS to guide recovery-oriented discussions has been suggested as one way in which this shift could be supported [36]. However, even though the RAS-DS was specifically designed with the intention to support greater self-determination and shared decision making [26], there are clearly barriers to this aspect of ‘implementation’. Many services only use RAS-DS as an outcome measure without taking up the additional opportunities to focus on person-centred planning and shared decision making. Implementation science involves understanding and addressing the barriers and enablers to systematic uptake of evidence-based practices [37]. Respondents in this study provided valuable insights into these barriers.

More than half of all respondents had some level of difficulty interpreting and discussing scores with service users and then ‘translating’ that discussion into a collaborative recovery-focused action plan. It was therefore unsurprising that training and support was one of the most frequently mentioned factors that had enabled, or could enable, people to use the RAS-DS as a goal planning tool. This need was also apparent in data about barriers to its use. Service users providing ‘unreliable’ responses, finding the RAS-DS stressful and or struggling to understand the RAS-DS are all more likely be concerns in situations where service users complete the RAS-DS without support or discussion. These issues would be common across many outcome measurement tools. Yet in skilled and sensitive discussion, each of these could be a basis for developing a deeper mutual understanding of the service user’s needs and desires. Rather than being ‘unreliable’, unexpected responses to certain questions may be an opportunity to develop stronger shared understanding between the service user and their worker. They could also uncover that the service user is uninterested in the RAS-DS, suggesting that alternative methods for planning may be more beneficial. Inattentive responses could trigger a discussion of what alternative topics or exercises would be more valued and meaningful. If a service user finds questions about a particular area of life upsetting, then this opens the opportunity for caring, trauma-informed discussions to occur. This upset may well indicate that an area is particularly meaningful to them and may benefit from attention. Difficulty understanding a question is an opportunity for explanation and dialogue. Flexible and person-centred use of RAS-DS can also address some concerns with its use in specific populations. For example, it could be initially self-completed or administered via interview, depending on the person, and could be completed in multiple sittings for people with negative symptoms or cognitive issues.

Helping practitioners to develop the confidence and skills to use these options and openings is likely to require provision of accessible training resources and structured guidance. In line with recovery-oriented principals of service-user engagement and the co-design history of the RAS-DS itself, any training resources should be created using a co-design approach [38, 39]. Co-design involves designing with people rather than for them [40]. End users are central to design, development and testing [38, 39] and this helps to ensure that resources meet the end users’ needs.

In response to these findings, our team have embarked on a range of initiatives. First, we have co-designed new print and video resources to assist staff to successfully engage with service-users around the RAS-DS and to set the tone for individualised recovery conversations. These are freely available online (https://ras-ds.net.au/resources). We are currently embarking on a co-design process to create a digital app that will address a range of the needs identified. While only a minority of respondents explicitly suggested digitalisation of the RAS-DS as a support idea, the planned platform will bring benefits beyond automating the scoring of RAS-DS. It will enable easier ‘on-the-spot’ discussion of the results, and facilitate the comparison of scores over time. The digital resource will have built in features to facilitate staff to initiate person-centred discussions, based on the results from the RAS-DS, and support service users to take a greater leadership role within that collaboration when developing recovery-focused action plans.

Beyond supporting individual mental health workers, however, it is clear from our findings that addressing the organisational context is also critical. Many participants who provided textual responses to open-ended questions identified contextual rather than personal barriers to using RAS-DS as a goal planning tool. These contextual barriers included organisational policies and lack of organisational support (34%), lack of time (22%) and lack of colleagues’ awareness of RAS-DS (20%). These results suggest that work is required to elevate the organisational or management understanding of and valuing of the opportunity that RAS-DS presents to their service and those accessing it.

This echoes the learnings from the implementation science field – the need to focus beyond the individual worker or service-user level to identify and address organisational and policy level barriers to systemic implementation [41]. Only with leaders championing the use of RAS-DS as more than an outcome measure will workers be afforded the time and support they need to engage with it more deeply with service-users. Addressing organisational barriers is complex but important for long term sustainability. Easily digestible, evidence-based materials that staff and other advocates could use to explain the benefits of the collaborative use of RAS-DS to managers and colleagues may be a starting point.

### Limitations

As with any convenience sample, readers should interpret the findings with consideration for the sample. The sampling frame included people who had previously contacted the researchers due to their interest in RAS-DS, suggesting that they may be a motivated and recovery-oriented group. Further, the 21% of this population who chose to respond to the survey may be more invested in the use of RAS-DS than non-responders. Thus, our data may overestimate the use of RAS-DS as a goal planning tool amongst RAS-DS users more generally. Nevertheless, the findings provide insights into the circumstances and supports needed to enable existing and intending users of RAS-DS to more fully utilise its potential to promote recovery-based practice.

In summary, while RAS-DS is used internationally as a tool to measure recovery, barriers exist to its systematic use as a tool to facilitate recovery through greater service-user leadership in their own recovery action planning. Future work, informed by implementation science wisdom and adopting a co-design approach, is required to better realise this potential.

## Data Availability

The dataset generated during the current study are not publicly available due to the possibility of identifiability of individuals or organisations from qualitative data. All enquiries about the dataset should be directed to anne.honey@sydney.edu.au.

## Abbreviations

RAS-DS: Recovery Assessment Scale: Domains and Stages
